# Congenital Cytomegalovirus and Hearing Loss: The State of the Art

**DOI:** 10.3390/jcm12134465

**Published:** 2023-07-03

**Authors:** Mirko Aldè, Sandro Binda, Valeria Primache, Laura Pellegrinelli, Elena Pariani, Fabrizio Pregliasco, Federica Di Berardino, Giovanna Cantarella, Umberto Ambrosetti

**Affiliations:** 1 Department of Clinical Sciences and Community Health, University of Milan, 20122 Milan, Italy; federica.diberardino@unimi.it (F.D.B.); giovanna.cantarella@unimi.it (G.C.); umberto.ambrosetti@unimi.it (U.A.); 2 Audiology Unit, Department of Specialist Surgical Sciences, Fondazione IRCCS Ca’ Granda Ospedale Maggiore Policlinico, 20122 Milan, Italy; 3 Department of Biomedical Sciences for Health, University of Milan, 20133 Milan, Italy; sandro.binda@unimi.it (S.B.); valeria.primache@unimi.it (V.P.); laura.pellegrinelli@unimi.it (L.P.); elena.pariani@unimi.it (E.P.); fabrizio.pregliasco@unimi.it (F.P.); 4 Otolaryngology Unit, Department of Specialist Surgical Sciences, Fondazione IRCCS Ca’ Granda Ospedale Maggiore Policlinico, 20122 Milan, Italy

**Keywords:** congenital cytomegalovirus, hearing loss, single-sided deafness, cochlear implantation, vaccine

## Abstract

In developed countries, congenital cytomegalovirus (cCMV) infection is the most common congenital viral infection, representing the leading non-genetic cause of sensorineural hearing loss (HL). Diagnosis of cCMV infection can be performed by detection of CMV DNA in urine or saliva within 2–3 weeks after birth, or later in dried blood samples on the Guthrie card. Currently, there are many controversies regarding the preventive, diagnostic, and therapeutic approaches to cCMV infection. HL secondary to cCMV is highly variable in onset, side, degree, audiometric configuration, and threshold changes over time. Therefore, it is of paramount importance to perform a long and thorough audiological follow-up in children with cCMV infection to ensure early identification and prompt treatment of progressive and/or late-onset HL. Early cochlear implantation appears to be a valid solution not only for children with bilateral profound HL, but also for those with single-sided deafness, improving localization ability and understanding speech in noisy environments. Moreover, the decision to apply a unilateral cochlear implant in children with cCMV is strengthened by the non-negligible possibility of hearing deterioration of the contralateral ear over time.

## 1. Introduction

Human cytomegalovirus (CMV), also known as human herpesvirus 5 (HHV-5), is a member of the Herpesviridae family and belongs to the Betaherpesvirinae subfamily [1,2]. The Herpesviridae family also includes other eight viruses that primarily infect humans: herpes simplex virus-1 (HHV-1), herpes simplex virus 2 (HHV-2), and varicella zoster virus (HHV-3), belonging to the Alphaherpesvirinae subfamily; Epstein–Barr virus (HHV-4) and Kaposi’s sarcoma-associated herpesvirus (HHV-8), belonging to the Gammaherpesvirinae subfamily; and roseolovirus (HHV-6A, HHV-6B, and HHV-7) belonging to the Betaherpevirinae subfamily [1]. CMV is characterized by a large double-stranded DNA genome and is considered the most complex herpesvirus [2]. The name is derived from the Greek words “cyto”, which means “cell”, and “megalo”, which means “big”. Indeed, CMV causes cyto-nucleomegaly and classic “owl’s eye” inclusions on histology [2]. These peculiar intranuclear inclusions were first detected in 1881 by Ribbert [3], while Goodpasture and Talbot in 1921 were the first to suggest that the “cytomegaly” could be due to a viral agent [4]. Human CMV was first isolated independently by Smith, Rowe, and Weller between 1956 and 1957, while the term “cytomegalovirus” was first proposed in 1960 by Weller [5].

CMV infection is ubiquitous, does not show seasonal variations, and is relatively common among women of reproductive age, with seroprevalence ranging from 45 to 100% [6]. Active CMV infection can result from either primary or non-primary infection. Primary infection occurs when an individual without immunity against CMV becomes infected for the first time, while non-primary infection is due to reinfection with exogenous CMV strains or reactivation of latent endogenous CMV [7]. Indeed, CMV resides latently in cells of the myeloid compartment, including CD34^+^ hematopoietic progenitor cells and circulating monocytes, and can occasionally reactivate, especially in immunocompromised patients or during critical illness [8]. CMV infection can be transmitted from one individual to another (“horizontal transmission”) through direct contact with body fluids (including saliva, urine, tears, genital secretions, organ transplant, or blood transfusion), or from mother to child (“vertical transmission”) through the placenta (“congenital CMV (cCMV) infection”), delivery, or breast milk (“postnatal CMV infection”) [9,10]. CMV infection is generally asymptomatic or may present as a mononucleosis syndrome in healthy people, while it is potentially life-threatening in immunocompromised patients [9].

In developed countries, cCMV infection is the most common congenital viral infection, with an overall birth prevalence of approximately 0.6% [9,11]. Young children represent the main source of transmission to pregnant women because they shed CMV in urine and saliva at high viral load over a considerable period of time [12]. It is estimated that 1–4% of CMV seronegative mothers will become infected during pregnancy, while approximately 10–30% of women with preconception immunity will experience non-primary infection [11]. The risk of vertical transmission after primary maternal CMV infection increases with gestation age, ranging from 20% in the first trimester to 75% in the third trimester, but the risk of severe fetal damage decreases with increasing gestational age [10]. Overall, the risk of vertical transmission after non-primary infection is much lower (approximately 1–3%) [11]. Approximately 10% of neonates with cCMV infection have symptoms at birth [13]. Disease manifestations can range from mild non-specific findings (e.g., petechiae, thrombocytopenia, anemia, leukopenia, conjugated hyperbilirubinemia, mild hepatosplenomegaly, or small for gestational age (SGA)) to moderately and severely symptomatic cCMV infections defined by the presence of multiple manifestations (e.g., widespread petechiae, jaundice, and marked hepatosplenomegaly) and/or central nervous system involvement (e.g., microcephaly, ventriculomegaly, periventricular cysts, and cerebral or cerebellar hypoplasia) or life-threatening disease [14]. Both asymptomatic and symptomatic newborns with cCMV are at risk of developing long-term neurodevelopmental disorders, such as sensorineural hearing loss (SNHL), visual impairment, cerebral palsy, autism spectrum disorder, and intellectual disability [13,15]. Specifically, permanent sequelae are observed in about 40–60% of infants with symptomatic cCMV infection and 10–15% of infants with asymptomatic cCMV infection [16]. Congenital CMV infection is currently estimated to be the leading non-genetic cause of SNHL [17]. Conversely, postnatal CMV infection is not recognized as a direct cause of SNHL in the pediatric population [18,19].

The aim of this narrative review is to provide a comprehensive and critical overview regarding cCMV, focusing on management controversies and cCMV-related hearing loss (HL). We screened titles, abstracts, and full texts from relevant literature to evaluate the content of the articles and extract valuable information. We also reported the experience of our tertiary-level hearing referral center with CMV-infected children.

## 2. Current Controversies in Preventive, Diagnostic, and Therapeutic Approaches to Congenital Cytomegalovirus Infection

### 2.1. Awareness and Knowledge of Congenital Cytomegalovirus Infection

Congenital CMV is a silent global burden that remains largely unrecognized in infants due to the high prevalence of non-specific symptoms or asymptomatic patients [20]. It has been widely demonstrated that women have poor knowledge of cCMV infection compared to other less frequent congenital diseases, such as Down syndrome, toxoplasmosis, and human immunodeficiency virus (HIV) infection [21,22,23]. Given the poor awareness and knowledge of cCMV infection among the world’s population and the lack of targeted prevention strategies, cCMV can be considered “an elephant in our living room” [24]. Providing proper prenatal education aimed at increasing awareness and knowledge of cCMV infection among pregnant women is a need highlighted by studies conducted in several countries, including the United States [25], Canada [26], Japan [22], Australia [27], Saudi Arabia [23], Germany [28], France [29], Switzerland [21], Italy [30], Spain [31], Portugal [32], the United Kingdom [33], and the Republic of Ireland [34]. Moreover, it is crucial to increase awareness of cCMV infection also among the male population and healthcare providers. In particular, men can serve as a vector for maternal infection and should be educated about behaviors that reduce the risk of CMV transmission, such as using condoms during sexual intercourse [30]. Similarly, healthcare professionals should play a key role in raising awareness among pregnant women through the dissemination of appropriate information [35]. However, their knowledge about cCMV is often low, negatively impacting prenatal counseling [31,36]. Indeed, most women are unaware of cCMV or how to reduce the risk of infection during pregnancy, in part due to poor health professionals’ awareness [37]. In this context, it is necessary to promote health campaigns and health education strategies to enhance awareness about cCMV infection [31]. All pregnant women should be adequately advised to avoid certain behavioral practices that increase the risk of CMV infection, such as kissing a child on the lips, sharing utensils, changing diapers, and practicing unsafe sex [10]. As a matter of fact, because no vaccine is currently available and treatment options are limited, the main measures to prevent CMV infection during pregnancy are based on rigorous respect of the hygienic-behavioral rules, such as handwashing after exposure to young children’s body fluids [10,30].

### 2.2. Maternal Serological Screening for Cytomegalovirus Infection

Diagnosis of primary CMV infection during pregnancy can be made by detection of CMV IgG seroconversion or CMV IgM positivity associated with low IgG avidity [38]. Currently, most public health policies and international scientific societies do not routinely recommend universal maternal prenatal screening for CMV infection due to the following reasons [30,39,40,41]: (a) lack of highly sensitive and specific prenatal tests; (b) CMV serological screening is not applicable to non-primary CMV infection; (c) lack of effective interventions to prevent transmission to the fetus; (d) lack of safe and effective prenatal treatments; (e) inability of laboratory tests to predict which babies will develop long-term neurological and audiological complications; (f) potentially increased rate of unnecessary abortions. Moreover, the false-positive rate would be much higher in the screened population than in women preselected for suspected CMV infection and, as a result, many pregnant women would undergo unnecessary additional testing and invasive procedures [39,42]. Therefore, according to the general agreement, CMV serologic testing should be offered only to pregnant women with symptoms and/or signs suggestive of primary CMV infection (e.g., influenza-like illness and/or fetal abnormalities on prenatal ultrasound examination) [42]. However, most pregnant women want to have CMV serological screening once informed about cCMV infection [43]; some of them make use of the maternal serological screening even if it is not recommended by the national guidelines [28,30,38,44,45]. As a matter of fact, maternal CMV screening followed by targeted neonatal CMV screening (testing neonates whose mothers become seropositive during pregnancy) may help identify asymptomatic cCMV cases in an early stage; Naessens et al. reported that this type of screening allowed the detection of 82% of all cCMV infections [46]. Moreover, women who are aware that they are susceptible to primary CMV infection based on serology are more likely to practice hygiene measures [45]. It is also important to highlight that maternal administration of oral valaciclovir following maternal primary infection in the first trimester of pregnancy has been demonstrated to be effective in reducing the rate of fetal CMV infection [47]. Therefore, maternal CMV screening followed by valaciclovir prevention may prevent most severe cases of cCMV infection [40]. However, possible adverse effects, especially acute renal failure, should be taken into consideration in pregnant women taking valaciclovir. Further studies are needed to investigate the safety and effectiveness of prenatal valaciclovir therapy in pregnancies with maternal CMV infection [48].

### 2.3. Neonatal Screening for Congenital Cytomegalovirus Infection

Diagnosis of cCMV infection can be performed by detection of CMV DNA in urine or saliva samples within 2–3 weeks after birth, or later by polymerase chain reaction (PCR) assay of dried blood spot (DBS) samples on the Guthrie card, which are universally collected within 3 days from birth [49,50,51]. Targeted cCMV screening through saliva PCR in children who fail the universal newborn hearing screening (UNHS) has been demonstrated to fall within the range between cost-neutral and cost-saving [52]. As a matter of fact, this cCMV screening method is feasible and acceptable to parents, providing the opportunity to start the treatment with oral valganciclovir within the first month of life [52,53]. However, performing cCMV neonatal screening only in babies who fail UNHS would not identify asymptomatic CMV-infected children who will develop late-onset HL [54]. A large study by Fowler et al. has shown that 43% of infants with CMV-related SNHL in the neonatal period and cCMV infants who are at risk of late-onset SNHL were not identified by UNHS [55]. Therefore, new strategies to identify all children with cCMV who remain at risk of late-onset and progressive HL are of paramount importance. In this context, it may be beneficial to extend targeted cCMV screening to children who pass UNHS but are at increased risk of cCMV infection, such as those born SGA or with microencephaly [54]. Indeed, an expanded targeted early cCMV testing program has been shown to improve detection rates of symptomatic cCMV cases, and should be considered as a valid alternative approach to hearing-targeted CMV testing [56]. In particular, cCMV infection should be suspected in all newborns: (a) whose mothers became CMV-seropositive during pregnancy; (b) who have received a confirmed diagnosis of SNHL; (c) who have signs and symptoms suggestive of CMV infection (e.g., SGA, microcephaly, petechiae, thrombocytopenia, unexplained hepatosplenomegaly, idiopathic elevated liver enzymes, and jaundice); (d) who have abnormal neuroimaging consistent with CMV infection (e.g., periventricular calcifications, ventriculomegaly, subependymal pseudocysts, white matter changes, cerebral or cerebellar hypoplasia, and lenticulostriate vasculopathy) [14,57].

In the case of late-onset HL, PCR analysis of DBS samples on the Guthrie card can help confirm or rule out cCMV infection even months or years after birth, allowing for adequate audiological follow-up [49,50,51]. As a matter of fact, DBS are the only samples routinely collected at birth and stored for a long time (which varies according to the policies of each country), representing a “universal newborns biobank” that may offer the best chance to obtain a retrospective cCMV diagnosis [58].

A recent prospective multicenter study has confirmed that without universal newborn CMV screening, some infected infants who develop late neurological and audiological sequelae may go unrecognized [59]. Indeed, thanks to improved diagnosis and treatments, a universal neonatal CMV screening would meet the minimum criteria to be included as a primary target condition in a newborn screening program (i.e., it can be identified at a period of time at which it would not ordinarily be clinically detected, a test with appropriate sensitivity and specificity is available, there are demonstrated benefits of early detection, there are timely intervention and efficacious treatment, and the benefits outweigh the costs and potential harms) [60]. In particular, universal newborn CMV screening would be essential to identify asymptomatic or mild symptomatic CMV-infected babies who pass UNHS, ensuring proper management, treatment, and follow-up [59,60].

### 2.4. Antiviral Therapy for Congenital Cytomegalovirus Infection

According to the general agreement, treatment with antiviral drugs should be considered only for neonates with severely/moderately symptomatic cCMV infection at birth [14,61]. Intravenous ganciclovir and its orally available prodrug, valganciclovir, are the first-line antiviral agents of choice [62]. Newborns with non-life-threatening disease are generally treated with oral valganciclovir; the recommended dose is 16 mg/kg/dose, administered twice daily for a total of 6 months [63]. Antiviral therapy should be started as soon as a virologic positive result is available, being effective in improving hearing and neurodevelopmental outcomes if started within the first month of life [62]. However, some studies have demonstrated the benefits and safety aspects of treating children with cCMV even beyond the recommended neonatal period [64,65]. Oral valganciclovir appears to have a beneficial role in both preventing and improving SNHL in children with symptomatic cCMV infection [66,67]. Although prolonged valganciclovir treatment for cCMV is generally safe and well-tolerated, a close monitoring of the white blood cell count and hemoglobin levels is mandatory; in particular, severe neutropenia (absolute neutrophil < 500/μL) is a possible adverse effect that can lead to treatment discontinuation [68]. Some retrospective studies have suggested that even children with isolated SNHL due to cCMV may benefit from valganciclovir [30,69,70], but to date no specific clinical trial data are available to support the routine use of antiviral treatment in these patients [71]. However, a study conducted in the United States has shown that the proportion of infants with cCMV treated with valganciclovir has increased markedly in recent years for all disease severity groups, including those without clinical findings [72]. It is crucial to point out that the use of valganciclovir may be associated with many serious adverse effects, such as neutropenia, anemia, thrombocytopenia, hepatotoxicity, and nephrotoxicity [63,68]. Thus, although many experts encourage the use of valganciclovir in infants with isolated HL [14], shared decision-making between pediatricians and parents is extremely important due to the sparse data on benefits and the known risks of treatment [71].

Thanks to animal studies focusing on antivirals and CMV-related SNHL, new and unexpected treatment options may be available in the near future. Using a murine model of CMV infection during auditory development, Sun et al. have recently shown that peripheral infection of newborn mice with murine CMV results in focal infection of the cochlea and virus-induced cochlear inflammation [73]. Cyclic cidofovir has also been demonstrated to prevent CMV-induced SNHL and associated cochlear histologic changes in guinea pigs [74].

### 2.5. Vaccines against Congenital Cytomegalovirus Infection

A vaccine against cCMV infection is a major public health priority. Although the development of CMV vaccines began in the 1970s [75,76], no CMV vaccine is available for humans so far [77]. In the year 2000, the National Academy of Medicine of the United States published a document that assigned the human CMV vaccine a high development priority [78]. As a matter of fact, the development of a vaccine capable of conferring effective protection against primary CMV infection may be a valid solution to reduce the serious consequences of intrauterine CMV infection. However, it should be emphasized that a strategy aimed at preventing only primary maternal infection would not address HL and other severe neurological sequelae secondary to cCMV in children born to mothers with pre-existing CMV immunity [79]. Indeed, maternal non-primary CMV infection during pregnancy is considered the greatest burden due to the high seroprevalence of CMV among women of childbearing age [9,30]. Therefore, ideal vaccines should have the ability not only to protect seronegative women from primary infection but also to enhance the immune response in seropositive women to prevent reactivation or reinfection [80]. A viable solution might be a recombinant CMV glycoprotein B vaccine with MF59 adjuvant that appeared to boost both antibody and CD4 T-cell responses in previously CMV-seropositive women, thus raising the possibility to prevent vertical transmission [81]. Unfortunately, CMV vaccines evaluated in clinical trials so far have demonstrated only about 45–50% efficacy against CMV primary infection and none of these have been approved [82,83]. However, a recent mathematical model by Byrne et al. has predicted that even modestly protective CMV vaccination of young children would significantly reduce both CMV transmission to pregnant women and the prevalence of cCMV [84]. Currently, several CMV vaccines are under development, based on different strategies: live-attenuated and disabled-infectious single-cycle vaccines, adjuvanted recombinant protein vaccines, DNA vaccines, messenger RNA (mRNA) vaccines, virus-like particle vaccines, viral vectored vaccines, and peptide vaccines [79]. One of the most promising approaches seems to be the expression of several antigens within a single vaccine, which could be ensured by vector vaccines and mRNA vaccines, thus stimulating more areas of the immune system [85]. In particular, Moderna’s vaccine candidate against CMV, mRNA-1647, combines six mRNAs in one vaccine and demonstrated functional antigen-specific responses in phase 1 and 2 studies without causing serious side effects [86]. The phase 3 study, known as CMVictory, is currently evaluating the safety, efficacy, and immunogenicity of mRNA-1647 against primary CMV infection in women aged 16–40 years [86]. After 50 years of failed attempts to develop a licensed CMV vaccine, modern platforms for vaccine construction finally allow hope for an effective prophylactic [77].

## 3. Hearing Loss Associated with Congenital Cytomegalovirus Infection

The association between cCMV infection and HL was first described in 1964 [87]. Although many studies have been conducted on this important public health issue since then, cCMV infection remains the leading non-genetic cause of SNHL in children in the developed world [16,17,62,88]. Congenital CMV is estimated to be responsible for HL in one in five hearing-impaired children with no other known risk factors [89]. A possible pathogenetic hypothesis is that CMV infection of the marginal cell layer of the stria vascularis may alter potassium and ion circulation, dissipating the endocochlear potential with consequent degeneration of the hair cells of the organ of Corti [90]. Paradoxically, hair cells appear to be spared by CMV infection [89].

The characteristic of HL due to cCMV infection are extremely variable [16,17,88]. First, HL is typically sensorineural and can occur in both symptomatic and asymptomatic children [16,17,88]. Although HL in asymptomatic children (“isolated HL”) is often considered a distinct category, some European experts classify infants with isolated SNHL as having severely symptomatic cCMV [14,71]. SNHL can be present at birth (“congenital HL”) or appear sometime later in life (“delayed-onset HL”) [16,17,88]. A recent systematic review by Vos et al. has reported that the prevalence of HL at birth is over 33% among symptomatic CMV-infected newborns and less than 15% in asymptomatic infections [88]. SNHL presents with later onset in about 10–20% of cCMV cases [57]. The hearing threshold may fluctuate or deteriorate over time [16,17,88]. Fluctuating HL is typically not explained by concurrent middle ear infections and may occur at only a few frequencies [16]. Hearing threshold deterioration can be observed in about half of children with SNHL at birth, regardless of whether they have asymptomatic or symptomatic infection [16]. However, in children with symptomatic CMV infection, HL is generally more severe and tends to progress earlier [16,57]. It is important to underline that a diagnosis of cCMV does not exclude the coexistence of other disorders that might explain hearing deterioration, such as genetic syndromes, specific mutations in genes associated with isolated HL, or ear malformations [30]. According to Peterson et al., the sub-cohort of CMV-positive newborns with symmetric mild-to-moderate bilateral HL will have at least a 7% chance of having pathogenic gene variants associated with HL [91]. Therefore, children with bilateral symmetric SNHL should undergo comprehensive genetic testing, regardless of cCMV status [91].

Delayed-onset and progression of SNHL among children with asymptomatic cCMV infection continue to occur throughout adolescence [92]. However, in these patients the risk of developing HL after 5 years of age is not significantly increased compared to uninfected children [92]. SNHL secondary to cCMV can affect one or both ears [16,17,88]. Most symptomatic children have bilateral HL, whereas unilateral HL predominates among asymptomatic children [17]. A study by Torrecillas et al. has demonstrated that in most children with cCMV infection and isolated SNHL, the poorer-hearing ear worsens earlier and more precipitously than the better-hearing ear [93]. HL can also have different audiometric configurations (rising, flat, or sloping) and range from mild to a profound degree [16,17,88]. A thorough audiological follow-up is recommended for all children with cCMV infection due to the possible late-onset, progressive, and fluctuating nature of HL: diagnostic evaluations should be performed every 3–6 months for the first year of life, then every 6 months until 3 years of age, and annually until 6 years of age [57]. A primary aim of this long-term audiological follow-up is to ensure timely diagnosis and reduce the possibility of unaddressed HL, which has a negative impact not only on global costs but also on children’s language, attention, emotions, and behavior [19,57]. All children diagnosed with SNHL should be promptly fitted with appropriate hearing aids; however, for children with severe to profound HL, hearing aids may be insufficient for HL rehabilitation, and cochlear implantation should be considered [57]. Lanzieri et al. have reported that approximately 2% of children with asymptomatic cCMV infection develop severe enough SNHL to meet cochlear implantation candidacy [92]. Several studies have shown that cochlear implants in children with cCMV generally have positive long-term outcomes, although the effectiveness could be lower in children with severe neurological sequelae [94,95,96]. Clinicians should take into account the negative effects of comorbidities associated with cCMV in the rehabilitation phase of cochlear implantation, explaining to parents that cochlear implant performance may be delayed [95]. A recent study by Cushing et al. has found that auditory nerve and brainstem responses to initial cochlear implant stimulation are similar in children with cCMV-related SNHL compared with GJB2-related SNHL [97]. Bolduc et al. have also suggested that antiviral treatment of cCMV appears to be a significant prognostic factor for the auditory progression of children with cochlear implantation [98]. Congenital CMV is one the most common causes of single-sided deafness (SSD) and represents a major predictor of acceptability of unilateral cochlear implantation in children due to the risk of progressive deterioration in the better-hearing ear [99]. In recent years, the Food and Drug Administration (FDA) has approved cochlear implantation for children aged 5 years and older with SSD: MED-EL received the first approval in 2019, while Cochlear Americas received it in 2022 [100]. However, auditory neuroplasticity is known to be greatly reduced after 7 years of age, implying only a 2-year window of potential benefit for children with congenital SSD [101]. As a matter of fact, if children with SSD do not receive a cochlear implant until they exhibit poor performance in elementary school, the window of maximal neuroplasticity may have passed [101]. A recent prospective clinical trial demonstrated that children implanted for unilateral HL have significant improvements in speech perception in quiet conditions, speech perception in noisy conditions, and localization abilities [102]. In CMV-infected children with SSD, early unilateral cochlear implantation may [99,103,104]: (a) rapidly restore bilateral auditory input to the cortex needed to improve binaural hearing; (b) serve as a lifeline in case of rapidly progressive HL in the normal ear; (c) improve auditory sensitivity in symptomatic children with severe visual impairment. Thus, it is clearly advantageous to implant the SSD ear rather than waiting for the contralateral ear to decline [100].

## 4. Our Audiological Experience with Children Affected by Congenital Cytomegalovirus Infection

Many children who receive a diagnosis of cCMV infection from different hospitals of the Metropolitan City of Milan (Italy) are referred to our tertiary-level hearing referral center in the Fondazione IRCCS Ca’ Granda, Ospedale Maggiore Policlinico (Milan, Italy). According to our current assessment protocol, all children diagnosed with cCMV infection undergo otomicroscopy, tympanometry, reflex threshold measurements, behavioral audiometry, click-evoked auditory brainstem responses (ABRs), and tone burst-evoked ABRs once every 3 months up to 1 year of age, once every 6 months from 1 to 3 years of age, once a year from 3 to 6 years of age, and as quickly as possible if hearing deterioration is suspected. Moreover, all CMV-infected children with confirmed SNHL are referred to an experienced clinical geneticist for further evaluation, including next-generation sequencing (NGS) to exclude coexisting pathogenic mutations. At the first diagnosis of SNHL of unknown etiology, we also request the Virology Laboratory of the Department of Biomedical Sciences for Health of the University of Milan to rule out cCMV infection. Indeed, this virology laboratory has developed a molecular method for the retrospective diagnosis of cCMV infection based on the identification of viral DNA in DBS of newborns on the Guthrie card by PCR, with a sensitivity and specificity (obtained by comparison with the reference diagnostic test by viral isolation in cell culture from urine samples collected within the first 2–3 weeks of life) greater than 99% [49,51]. In brief, this method consists of DNA extraction from the DBS samples by heat shock, followed by amplification of a specific sequence of the viral genome using a nested PCR [49,51]. In accordance with the Italian law, DBS samples are routinely collected within 3 days from the birth and used for the early diagnosis of hereditary genetic/metabolic diseases; the residual DBS material is stored at the corresponding regional screening center [51]. It is important to remember that in Northern Italy, not all hospitals perform targeted cCMV screening through saliva or urine PCR in children who fail the UNHS [51]. In this context, DBS testing may be useful in the retrospective diagnosis of cCMV, providing definitive diagnostic information about the etiology of SNHL [50,51,58]. In particular, we found that 6.1% of children referred to our pediatric outpatient audiology clinic, who had failed the UNHS and had not been screened for cCMV from their birth hospitals, subsequently received a diagnosis of cCMV infection thanks to DBS testing [51]. In a recent study conducted on 141 pediatric patients with cCMV infection, we have found that more than 1 in 5 children had a confirmed diagnosis of SNHL at the six-year audiological assessment; 40.6% of them were asymptomatic/mildly symptomatic at birth [30]. However, children with severely/moderately symptomatic cCMV infection had a higher prevalence of bilateral and severe-to-profound HL than children with asymptomatic/mild symptomatic cCMV infection [30]. Overall, hearing deterioration and threshold fluctuations over the years were observed in 11.3% and 27.7% of cases, respectively [30]. Moreover, 34.0% of children with cCMV had a diagnosis of speech-language delay, 12.8% of motor delay, 11.3% of balance disorders, and 4.3% of cognitive delay [30]. These findings highlight not only the importance of interdisciplinary evaluations with speech pathologists and pediatric neuropsychiatrists, but also the need of accurate vestibular assessment in all children with cCMV. Similar to HL, vestibular dysfunction in children with cCMV is highly variable: it can be unilateral or bilateral, mild to severe, stable or progressive, and early or delayed in onset [105,106]. In our audiologic center, vestibular function of CMV-infected children is routinely evaluated through bedside examination, video head impulse tests and static posturography. In particular, video head impulse tests are performed from the first year of age thanks to remote video recordings.

A recent systematic review by Shears et al. has suggested that CMV DBS testing should also be considered for children who present with vestibular dysfunction, balance problems and/or gross developmental delay [106]. Finally, in our experience, CMV-infected children with SSD should undergo cochlear implantation. Indeed, in absence of anatomical malformations or severe comorbidities, these children experience remarkable improvements in sound localization, speech understanding in noise, and quality of life within a few months of unilateral cochlear implant surgery [30].

## 5. Conclusions

Children diagnosed with cCMV, even if asymptomatic and with normal hearing at birth, should undergo a long and thorough audiological follow-up due to the risk of delayed-onset, progressive, and fluctuating HL. Currently, there are many controversies regarding the preventive (e.g., maternal prenatal screening for primary CMV infection is not routinely recommended), diagnostic (e.g., neonatal CMV screening is often offered only to babies who fail UNHS, and therefore children who develop late-onset HL due to cCMV infection go undetected), and therapeutic (e.g., oral valganciclovir is generally administered for 6 months only to symptomatic children, but could also have a role in preventing HL in asymptomatic children) approaches to cCMV infection. The characteristics of HL secondary to cCMV are highly variable in onset (at birth/late-onset), side (unilateral/bilateral), degree (mild to profound), audiometric configuration (rising/flap/sloping), and threshold changes over time (fluctuating, stable, sudden deterioration, progressive deterioration). All CMV-infected children with a confirmed diagnosis of SNHL should be promptly fitted with appropriate hearing aids; early cochlear implantation appears to be a valid solution not only for children with bilateral profound HL, but also for those with single-sided deafness, due to the risk of hearing deterioration over time. The development of a vaccine against human CMV is a public health priority that could finally reduce the burden of cCMV infection.

## Data Availability

Not applicable.

## References

[1] Davison A.J. (2010). Herpesvirus systematics. Vet. Microbiol..

[2] Louten J. (2022). Essential Human Virology.

[3] Ribbert D. (1904). Uber protozoenartige zellen in der niere eines syphilitischen neugoborenen und in der parotis von kindern. Zentralbl. Allg. Pathol..

[4] Goodpasture E.W., Talbot F.B. (1921). Concerning the nature of “proteozoan-like” cells in certain lesions of infancy. Am. J. Dis. Child..

[5] Riley H.D. (1997). History of the cytomegalovirus. South. Med. J..

[6] Cannon M.J., Schmid D.S., Hyde T.B. (2010). Review of cytomegalovirus seroprevalence and demographic characteristics associated with infection. Rev. Med. Virol..

[7] Griffiths P., Reeves M. (2021). Pathogenesis of human cytomegalovirus in the immunocompromised host. Nat. Rev. Microbiol..

[8] Smith N.A., Chan G.C., O’Connor C.M. (2021). Modulation of host cell signaling during cytomegalovirus latency and reactivation. Virol. J..

[9] Gupta M., Shorman M. (2022). Cytomegalovirus. StatPearls.

[10] Pass R.F., Anderson B. (2014). Mother-to-Child Transmission of Cytomegalovirus and Prevention of Congenital Infection. J. Pediatr. Infect. Dis. Soc..

[11] Swanson E.C., Schleiss M.R. (2013). Congenital cytomegalovirus infection: New prospects for prevention and therapy. Pediatr. Clin. N. Am..

[12] Davis N.L., King C.C., Kourtis A.P. (2017). Cytomegalovirus infection in pregnancy. Birth Defects Res..

[13] Boppana S.B., Ross S.A., Fowler K.B. (2013). Congenital cytomegalovirus infection: Clinical outcome. Clin. Infect. Dis..

[14] Luck S.E., Wieringa J.W., Blázquez-Gamero D., Henneke P., Schuster K., Butler K., Capretti M.G., Cilleruelo M.J., Curtis N., Garofoli F. (2017). Congenital Cytomegalovirus: A European Expert Consensus Statement on Diagnosis and Management. Pediatr. Infect. Dis. J..

[15] Korndewal M.J., Oudesluys-Murphy A.M., Kroes A.C.M., van der Sande M.A.B., de Melker H.E., Vossen A.C.T.M. (2017). Long-term impairment attributable to congenital cytomegalovirus infection: A retrospective cohort study. Dev. Med. Child. Neurol..

[16] Fowler K.B. (2013). Congenital cytomegalovirus infection: Audiologic outcome. Clin. Infect. Dis..

[17] Goderis J., De Leenheer E., Smets K., Van Hoecke H., Keymeulen A., Dhooge I. (2014). Hearing loss and congenital CMV infection: A systematic review. Pediatrics.

[18] Martinez-Gomez E., Perez-Carpena P., Flook M., Lopez-Escamez J.A. (2020). A Systematic Review on the Association of Acquired Human Cytomegalovirus Infection with Hearing Loss. J. Clin. Med..

[19] Aldè M., DIBerardino F., Marchisio P., Cantarella G., Iacona E., Ambrosetti U., Zanetti D. (2021). Sudden sensorineural hearing loss in children with dual positivity of serum anti-EBV IgM and anti-CMV IgM antibodies: A preliminary study. Minerva Pediatr..

[20] Manicklal S., Emery V.C., Lazzarotto T., Boppana S.B., Gupta R.K. (2013). The “silent” global burden of congenital cytomegalovirus. Clin. Microbiol. Rev..

[21] Willame A., Blanchard-Rohner G., Combescure C., Irion O., Posfay-Barbe K., Martinez de Tejada B. (2015). Awareness of Cytomegalovirus Infection among Pregnant Women in Geneva, Switzerland: A Cross-sectional Study. Int. J. Environ. Res. Public Health.

[22] Kobayashi M., Okahashi A., Okuyama K., Hiraishi N., Morioka I. (2021). Awareness and knowledge of congenital cytomegalovirus infection among pregnant women and the general public: A web-based survey in Japan. Environ. Health Prev. Med..

[23] Almishaal A.A. (2022). Knowledge of cytomegalovirus infection among women in Saudi Arabia: A cross-sectional study. PLoS ONE.

[24] Demmler-Harrison G.J. (2016). Congenital Cytomegalovirus Infection: The Elephant in Our Living Room. JAMA Pediatr..

[25] Tastad K.J., Schleiss M.R., Lammert S.M., Basta N.E. (2019). Awareness of congenital cytomegalovirus and acceptance of maternal and newborn screening. PLoS ONE.

[26] Wizman S., Lamarre V., Coic L., Kakkar F., Le Meur J.B., Rousseau C., Boucher M., Tapiero B. (2016). Awareness of cytomegalovirus and risk factors for susceptibility among pregnant women, in Montreal, Canada. BMC Pregnancy Childbirth.

[27] Lazzaro A., Vo M.L., Zeltzer J., Rawlinson W., Nassar N., Daly K., Lainchbury A., Shand A. (2019). Knowledge of congenital cytomegalovirus (CMV) in pregnant women in Australia is low, and improved with education. Aust. N. Z. J. Obstet. Gynaecol..

[28] Greye H., Henning S., Freese K., Köhn A., Lux A., Radusch A., Redlich A., Schleef D., Seeger S., Thäle V. (2022). Cross-sectional study to assess awareness of cytomegalovirus infection among pregnant women in Germany. BMC Pregnancy Childbirth.

[29] Alain S., Garnier-Geoffroy F., Labrunie A., Montané A., Marin B., Gatet M., Grosjean J., Dufour V., Saugeras M., Postil D. (2020). Cytomegalovirus (CMV) Shedding in French Day-Care Centers: A Nationwide Study of Epidemiology, Risk Factors, Centers’ Practices, and Parents’ Awareness of CMV. J. Pediatr. Infect. Dis. Soc..

[30] Aldè M., Caputo E., Di Berardino F., Ambrosetti U., Barozzi S., Piatti G., Zanetti D., Pignataro L., Cantarella G. (2023). Hearing outcomes in children with congenital cytomegalovirus infection: From management controversies to lack of parents’ knowledge. Int. J. Pediatr. Otorhinolaryngol..

[31] Castillo K., Hawkins-Villarreal A., Valdés-Bango M., Guirado L., Scazzocchio E., Porta O., Falguera G., López M., Palacio M., Gratacós E. (2022). Congenital Cytomegalovirus Awareness and Knowledge among Health Professionals and Pregnant Women: An Action towards Prevention. Fetal Diagn. Ther..

[32] Monteiro S., Gonçalves A., Torrão M.M., Costa V., Almeida A. (2023). Knowledge of cytomegalovirus and available prevention strategies in pregnancy: A cross-sectional study in Portugal. J. Matern.-Fetal Neonatal Med..

[33] Calvert A., Vandrevala T., Parsons R., Barber V., Book A., Book G., Carrington D., Greening V., Griffiths P., Hake D. (2021). Changing knowledge, attitudes and behaviours towards cytomegalovirus in pregnancy through film-based antenatal education: A feasibility randomised controlled trial of a digital educational intervention. BMC Pregnancy Childbirth.

[34] Basit I., Crowley D., Geary M., Kirkham C., Mc Dermott R., Cafferkey M., Sayers G. (2019). Awareness and Preventative Behaviours Regarding Toxoplasma, Listeria and Cytomegalovirus Among Pregnant Women. Ir. Med. J..

[35] Benou S., Dimitriou G., Papaevangelou V., Gkentzi D. (2022). Congenital cytomegalovirus infection: Do pregnant women and healthcare providers know enough? A systematic review. J. Matern.-Fetal Neonatal Med..

[36] Pesch M.H., Anderson C., Mowers E. (2020). Improving Obstetric Provider Congenital Cytomegalovirus Knowledge and Practices. Infect. Dis. Obstet. Gynecol..

[37] Midgley G., Smithers-Sheedy H., McIntyre S., Badawi N., Keogh J., Jones C.A. (2020). Congenital Cytomegalovirus Prevention, Awareness and Policy Recommendations—A Scoping Study. Infect. Disord. Drug Targets.

[38] Shimada K., Toriyabe K., Kitamura A., Morikawa F., Minematsu T., Ikejiri M., Suga S., Toyoda H., Amano K., Kitano M. (2021). Primary cytomegalovirus infection during pregnancy and congenital infection: A population-based, mother-child, prospective cohort study. J. Perinatol..

[39] Lazzarotto T., Blázquez-Gamero D., Delforge M.L., Foulon I., Luck S., Modrow S., Leruez-Ville M. (2020). Congenital Cytomegalovirus Infection: A Narrative Review of the Issues in Screening and Management From a Panel of European Experts. Front. Pediatr..

[40] Seror V., Leruez-Ville M., Ӧzek A., Ville Y. (2022). Leaning towards Cytomegalovirus serological screening in pregnancy to prevent congenital infection: A cost-effectiveness perspective. BJOG.

[41] Iijima S. (2022). Pitfalls in the Serological Evaluation of Maternal Cytomegalovirus Infection as a Potential Cause of Fetal and Neonatal Involvements: A Narrative Literature Review. J. Clin. Med..

[42] Pass R.F., Arav-Boger R. (2018). Maternal and fetal cytomegalovirus infection: Diagnosis, management, and prevention. F1000Research.

[43] Beaudoin M.L., Renaud C., Boucher M., Kakkar F., Gantt S., Boucoiran I. (2021). Perspectives of women on screening and prevention of CMV in pregnancy. Eur. J. Obstet. Gynecol. Reprod. Biol..

[44] Rubinacci V., Fumagalli M., Meraviglia G., Gianolio L., Sala A., Stracuzzi M., Dighera A., Zuccotti G.V., Giacomet V. (2022). Congenital CMV, Lights and Shadows on Its Management: The Experience of a Reference Center in Northern Italy. Children.

[45] Rudd I.P., Marzan M.B., Hui L. (2023). Cytomegalovirus serological screening at the first antenatal visit: A tertiary-centre audit of general practitioner practices and maternal seroprevalence. Aust. N. Z. J. Obstet. Gynaecol..

[46] Naessens A., Casteels A., Decatte L., Foulon W. (2005). A serologic strategy for detecting neonates at risk for congenital cytomegalovirus infection. J. Pediatr..

[47] Shahar-Nissan K., Pardo J., Peled O., Krause I., Bilavsky E., Wiznitzer A., Hadar E., Amir J. (2020). Valaciclovir to prevent vertical transmission of cytomegalovirus after maternal primary infection during pregnancy: A randomised, double-blind, placebo-controlled trial. Lancet.

[48] D’Antonio F., Marinceu D., Prasad S., Khalil A. (2023). Effectiveness and safety of prenatal valacyclovir for congenital cytomegalovirus infection: Systematic review and meta-analysis. Ultrasound Obstet. Gynecol..

[49] Binda S., Caroppo S., Didò P., Primache V., Veronesi L., Calvario A., Piana A., Barbi M. (2004). Modification of CMV DNA detection from dried blood spots for diagnosing congenital CMV infection. J. Clin. Virol..

[50] Meyer L., Sharon B., Huang T.C., Meyer L., Sharon B., Huang T.C., Meyer A.C., Gravel K.E., Schimmenti L.A., Swanson E.C. (2017). Analysis of archived newborn dried blood spots (DBS) identifies congenital cytomegalovirus as a major cause of unexplained pediatric sensorineural hearing loss. Am. J. Otolaryngol..

[51] Pellegrinelli L., Galli C., Primache V., Alde’ M., Fagnani E., Di Berardino F., Zanetti D., Pariani E., Ambrosetti U., Binda S. (2019). Diagnosis of congenital CMV infection via DBS samples testing and neonatal hearing screening: An observational study in Italy. BMC Infect. Dis..

[52] Gillespie A.N., Dalziel K., Webb E., Wong J., Jones C.A., Sung V., HearS-cCMV Project (2023). Targeted screening for congenital cytomegalovirus: A micro-costing analysis. J. Paediatr. Child Health.

[53] Fourgeaud J., Boithias C., Walter-Nicolet E., Kermorvant E., Couderc S., Parat S., Pol C., Mousset C., Bussières L., Guilleminot T. (2022). Performance of Targeted Congenital Cytomegalovirus Screening in Newborns Failing Universal Hearing Screening: A Multicenter Study. Pediatr. Infect. Dis. J..

[54] Aldè M., Ambrosetti U. (2023). Letter to the Editor. J. Paediatr. Child Health.

[55] Fowler K.B., McCollister F.P., Sabo D.L., Shoup A.G., Owen K.E., Woodruff J.L., Cox E., Mohamed L.S., Choo D.I., Boppana S.B. (2017). A Targeted Approach for Congenital Cytomegalovirus Screening within Newborn Hearing Screening. Pediatrics.

[56] Suarez D., Nielson C., McVicar S.B., Sidesinger M., Ostrander B., O’Brien E., Ampofo K., Ling C.Y., Miner L.J., Park A.H. (2023). Analysis of an Expanded Targeted Early Cytomegalovirus Testing Program. Otolaryngol. Head Neck Surg..

[57] Kettler M., Shoup A., Moats S., Steuerwald W., Jones S., Stiell S.C., Chappetto J. (2023). American Academy of Audiology Position Statement on Early Identification of Cytomegalovirus in Newborns. J. Am. Acad. Audiol..

[58] Pellegrinelli L., Alberti L., Pariani E., Barbi M., Binda S. (2020). Diagnosing congenital Cytomegalovirus infection: Don’t get rid of dried blood spots. BMC Infect. Dis..

[59] Chiereghin A., Pavia C., Turello G., Borgatti E.C., Baiesi Pillastrini F., Gabrielli L., Gibertoni D., Marsico C., De Paschale M., Manco M.T. (2022). Universal Newborn Screening for Congenital Cytomegalovirus Infection—From Infant to Maternal Infection: A Prospective Multicenter Study. Front. Pediatr..

[60] Lunardi S., Lorenzoni F., Ghirri P., Barría R.M. (2020). Universal screening for congenital CMV infection. Update on Critical Issues on Infant and Neonatal Care.

[61] Rawlinson W.D., Boppana S.B., Fowler K.B., Kimberlin D.W., Lazzarotto T., Alain S., Daly K., Doutré S., Gibson L., Giles M.L. (2017). Congenital cytomegalovirus infection in pregnancy and the neonate: Consensus recommendations for prevention, diagnosis, and therapy. Lancet Infect. Dis..

[62] Marsico C., Kimberlin D.W. (2017). Congenital Cytomegalovirus infection: Advances and challenges in diagnosis, prevention and treatment. Ital. J. Pediatr..

[63] Kimberlin D.W., Jester P.M., Sánchez P.J., Ahmed A., Arav-Boger R., Michaels M.G., Ashouri N., Englund J.A., Estrada B., Jacobs R.F. (2015). Valganciclovir for symptomatic congenital cytomegalovirus disease. N. Engl. J. Med..

[64] Dorfman L., Amir J., Attias J., Bilavsky E. (2020). Treatment of congenital cytomegalovirus beyond the neonatal period: An observational study. Eur. J. Pediatr..

[65] Morioka I., Kakei Y., Omori T., Nozu K., Fujioka K., Takahashi N., Yoshikawa T., Moriuchi H., Ito Y., Oka A. (2022). Oral Valganciclovir Therapy in Infants Aged ≤2 Months with Congenital Cytomegalovirus Disease: A Multicenter, Single-Arm, Open-Label Clinical Trial in Japan. J. Clin. Med..

[66] Bilavsky E., Shahar-Nissan K., Pardo J., Attias J., Amir J. (2016). Hearing outcome of infants with congenital cytomegalovirus and hearing impairment. Arch. Dis. Child..

[67] Suganuma E., Sakata H., Adachi N., Asanuma S., Furuichi M., Uejima Y., Sato S., Abe T., Matsumoto D., Takahashi R. (2021). Efficacy, safety, and pharmacokinetics of oral valganciclovir in patients with congenital cytomegalovirus infection. J. Infect. Chemother..

[68] Ziv L., Yacobovich J., Pardo J., Yarden-Bilavsky H., Amir J., Osovsky M., Bilavsky E. (2019). Hematologic Adverse Events Associated with Prolonged Valganciclovir Treatment in Congenital Cytomegalovirus Infection. Pediatr. Infect. Dis. J..

[69] Yilmaz Çiftdogan D., Vardar F. (2011). Effect on hearing of oral valganciclovir for asymptomatic congenital cytomegalovirus infection. J. Trop. Pediatr..

[70] Pasternak Y., Ziv L., Attias J., Amir J., Bilavsky E. (2018). Valganciclovir Is Beneficial in Children with Congenital Cytomegalovirus and Isolated Hearing Loss. J. Pediatr..

[71] Lanzieri T.M., Pesch M.H., Grosse S.D. (2023). Considering Antiviral Treatment to Preserve Hearing in Congenital CMV. Pediatrics.

[72] Leung J., Grosse S.D., Hong K., Pesch M.H., Lanzieri T.M. (2022). Changes in Valganciclovir Use Among Infants with Congenital Cytomegalovirus Diagnosis in the United States, 2009–2015 and 2016–2019. J. Pediatr..

[73] Sung C.Y.W., Seleme M.C., Payne S., Jonjic S., Hirose K., Britt W. (2019). Virus-induced cochlear inflammation in newborn mice alters auditory function. JCI Insight.

[74] White D.R., Choo D.I., Stroup G., Schleiss M.R. (2006). The effect of cidofovir on cytomegalovirus-induced hearing loss in a Guinea pig model. Arch. Otolaryngol. Head Neck Surg..

[75] Elek S.D., Stern H. (1974). Development of a vaccine against mental retardation caused by cytomegalovirus infection in utero. Lancet.

[76] (1975). Plotkin SA, Furukawa T, Zygraich N, Huygelen Candidate cytomegalovirus strain for human vaccination. Infect. Immun..

[77] Plotkin S.A. (2023). Can We Prevent Congenital Infection by Cytomegalovirus?. Clin. Infect. Dis..

[78] Stratton K.R., Durch J.S., Lawrence R.S., Institute of Medicine Committee to Study Priorities for Vaccine, D (2000). The national academies collection: Reports funded by national institutes of health. Vaccines for the 21st Century: A Tool for Decision Making.

[79] Sartori P., Egloff C., Hcini N., Vauloup Fellous C., Périllaud-Dubois C., Picone O., Pomar L. (2023). Primary, Secondary, and Tertiary Prevention of Congenital Cytomegalovirus Infection. Viruses.

[80] Chiopris G., Veronese P., Cusenza F., Procaccianti M., Perrone S., Daccò V., Colombo C., Esposito S. (2020). Congenital Cytomegalovirus Infection: Update on Diagnosis and Treatment. Microorganisms.

[81] Sabbaj S., Pass R.F., Goepfert P.A., Pichon S. (2011). Glycoprotein B vaccine is capable of boosting both antibody and CD4 T-cell responses to cytomegalovirus in chronically infected women. J. Infect. Dis..

[82] Pass R.F., Zhang C., Evans A., Simpson T., Andrews W., Huang M.L., Corey L., Hill J., Davis E., Flanigan C. (2009). Vaccine prevention of maternal cytomegalovirus infection. N. Engl. J. Med..

[83] Bernstein D.I., Munoz F.M., Callahan S.T., Rupp R., Wootton S.H., Edwards K.M., Turley C.B., Stanberry L.R., Patel S.M., Mcneal M.M. (2016). Safety and efficacy of a cytomegalovirus glycoprotein B (gB) vaccine in adolescent girls: A randomized clinical trial. Vaccine.

[84] Byrne C., Coombs D., Gantt S. (2022). Modestly protective cytomegalovirus vaccination of young children effectively prevents congenital infection at the population level. Vaccine.

[85] Scarpini S., Morigi F., Betti L., Dondi A., Biagi C., Lanari M. (2021). Development of a Vaccine against Human Cytomegalovirus: Advances, Barriers, and Implications for the Clinical Practice. Vaccines.

[86] Moderna Moderna Announces First Participant Dosed in Phase 3 Pivotal Registration Study of Its mRNA Cytomegalovirus (cmv) Vaccine. https://investors.modernatx.com/news/news-details/2021/Moderna-Announces-First-Participant-Dosed-in-Phase-3-Pivotal-Registration-Study-of-Its-mRNA-Cytomegalovirus-CMV-Vaccine/default.aspx.

[87] Medearis D.N. (1964). Viral infections during pregnancy and abnormal human development. Am. J. Obstet. Gynecol..

[88] Vos B., Noll D., Whittingham J., Pigeon M., Bagatto M., Fitzpatrick E.M. (2021). Cytomegalovirus-A Risk Factor for Childhood Hearing Loss: A Systematic Review. Ear Hear..

[89] Teissier N., Bernard S., Quesnel S., Van Den Abbeele T. (2016). Audiovestibular consequences of congenital cytomegalovirus infection. Eur. Ann. Otorhinolaryngol. Head Neck Dis..

[90] Gabrielli L., Bonasoni M.P., Santini D., Piccirilli G., Chiereghin A., Guerra B., Landini M.P., Capretti M.G., Lanari M., Lazzarotto T. (2013). Human fetal inner ear involvement in congenital cytomegalovirus infection. Acta Neuropathol. Commun..

[91] Peterson J., Nishimura C., Smith R.J.H. (2020). Genetic Testing for Congenital Bilateral Hearing Loss in the Context of Targeted Cytomegalovirus Screening. Laryngoscope.

[92] Lanzieri T.M., Chung W., Flores M., Blum P., Caviness A.C., Bialek S.R., Grosse S.D., Miller J.A., Demmler-Harrison G., Congenital Cytomegalovirus Longitudinal Study Group (2017). Hearing Loss in Children with Asymptomatic Congenital Cytomegalovirus Infection. Pediatrics.

[93] Torrecillas V., Allen C.M., Greene T., Park A., Chung W., Lanzieri T.M., Demmler-Harrison G. (2020). Should You Follow the Better-Hearing Ear for Congenital Cytomegalovirus Infection and Isolated Sensorineural Hearing Loss?. Otolaryngol. Head Neck Surg..

[94] Yoshida H., Takahashi H., Kanda Y., Kitaoka K., Hara M. (2017). Long-term Outcomes of Cochlear Implantation in Children with Congenital Cytomegalovirus Infection. Otol. Neurotol..

[95] Kraaijenga V.J.C., Van Houwelingen F., Van der Horst S.F., Visscher J., Huisman J.M.L., Hollman E.J., Stegeman I., Smit A.L. (2018). Cochlear implant performance in children deafened by congenital cytomegalovirus—A systematic review. Clin. Otolaryngol..

[96] Corazzi V., Ciorba A., Bianchini C., Rosignoli M., Negossi L., Minazzi F., Borin M., Malagutti N., Stomeo F., Pelucchi S. (2020). Outcome of cochlear implantation in children with congenital Cytomegalovirus infection: A retrospective case control study. Int. J. Pediatr. Otorhinolaryngol..

[97] Cushing S.L., Purcell P.L., Papaiaonnou V., Neghandi J., Daien M., Blaser S.I., Ertl-Wagner B., Wagner M., Sheng M., James A.L. (2022). Hearing Instability in Children with Congenital Cytomegalovirus: Evidence and Neural Consequences. Laryngoscope.

[98] Bolduc S.H., Bussières R., Philippon D., Côté M. (2021). The Correlation of Congenital CMV Infection and the Outcome of Cochlear Implantation. J. Int. Adv. Otol..

[99] Cushing S.L., Gordon K.A., Sokolov M., Papaioannou V., Polonenko M., Papsin B.C. (2019). Etiology and therapy indication for cochlear implantation in children with single-sided deafness: Retrospective analysis. HNO.

[100] Park L.R., Griffin A.M., Sladen D.P., Neumann S., Young N.M. (2022). American Cochlear Implant Alliance Task Force Guidelines for Clinical Assessment and Management of Cochlear Implantation in Children with Single-Sided Deafness. Ear Hear..

[101] Park L.R., Gagnon E.B., Brown K.D. (2021). The Limitations of FDA Criteria: Inconsistencies with Clinical Practice, Findings, and Adult Criteria as a Barrier to Pediatric Implantation. Semin. Hear..

[102] Brown K.D., Dillon M.T., Park L.R. (2022). Benefits of Cochlear Implantation in Childhood Unilateral Hearing Loss (CUHL Trial). Laryngoscope..

[103] Polonenko M.J., Gordon K.A., Cushing S.L., Papsin B.C. (2017). Cortical organization restored by cochlear implantation in young children with single sided deafness. Sci. Rep..

[104] Jenks C.M., Mithal L.B., Hoff S.R. (2021). Early Identification and Management of Congenital Cytomegalovirus. Otolaryngol. Clin. N. Am..

[105] Dhondt C., Maes L., Rombaut L., Martens S., Vanaudenaerde S., Van Hoecke H., De Leenheer E., Dhooge I. (2021). Vestibular Function in Children with a Congenital Cytomegalovirus Infection: 3 Years of Follow-Up. Ear Hear..

[106] Shears A., Yan G., Mortimer H., Cross E., Sapuan S., Kadambari S., Luck S., Heath P.T., Walter S., Fidler K.J. (2022). Vestibular and balance dysfunction in children with congenital CMV: A systematic review. Arch. Dis. Child.-Fetal Neonatal Ed..

